# Proteomic alterations in ovarian cancer—Predicting residual disease status using artificial intelligence and SHAP-based biomarker interpretation

**DOI:** 10.3389/fmed.2025.1562558

**Published:** 2025-07-23

**Authors:** Seyma Yasar, Rauf Melekoglu

**Affiliations:** ^1^Department of Biostatistics, and Medical Informatics, Medicine Faculty, Inonu University, Malatya, Türkiye; ^2^Department of Obstetrics and Gynecology, Faculty of Medicine, Inonu University, Malatya, Türkiye

**Keywords:** high-grade serous ovarian cancer (HGSOC), neoadjuvant chemotherapy (NACT), machine learning, proteomic biomarkers, SHAP analysis

## Abstract

**Introduction:**

High-grade serous ovarian cancer (HGSOC) is the most aggressive and prevalent subtype of ovarian Treatment outcomes are significantly influenced by residual disease status following neoadjuvant chemotherapy (NACT). Predicting residual disease before surgery can improve patient stratification and personalized treatment strategies.

**Methods:**

This study analyzed pre-NACT proteomic data from 20 HGSOC patients treated with NACT. Patients were categorized into two groups based on surgical outcomes: no residual disease (R0, *n* = 14) and suboptimal residual disease (R1, *n* = 6). From an initial set of 97 differentially expressed proteins, 18 significant proteins were selected using the BORUTA feature selection method. Three machine learning models-Random Forest (RF), Support Vector Machine (SVM), and Bootstrap Aggregation with Classification and Regression Trees (BaggedCART)-were developed and evaluated.

**Results:**

The Random Forest model achieved the best performance with an AUC of 0.955, accuracy of 0.830, sensitivity of 0.904, specificity of 0.763, and F1-score of 0.839. SHapley Additive exPlanations (SHAP) analysis identified five proteins (P48637, O43491, O95302, Q96CX2, and P49189) as the most influential predictors of residual disease. These proteins, including glutathione synthetase and peptidyl-prolyl cis-trans isomerase FKBP9, are associated with chemotherapy resistance mechanisms.

**Discussion:**

The findings demonstrate the potential of integrating proteomic data with machine learning techniques for predicting surgical outcomes in HGSOC. Identified protein signatures may serve as valuable biomarkers for anticipating NACT response and informing clinical decision-making, ultimately contributing to personalized patient care.

## 1 Introduction

Ovarian cancer is one of the deadliest gynecologic cancers and ranks as the eighth most common cancer among women worldwide. It is the third most common gynecologic cancer. Each year, approximately 300,000 new cases are detected worldwide, with approximately 200,000 deaths attributed to the disease (1). This type of cancer, which is more common in developed countries, North America, and Europe, poses a greater risk in women over the age of 50–60, those with a family history, and those who carry BRCA1/BRCA2 gene mutations (2). Pelvic examination, transvaginal ultrasonography, CA-125 blood tests, and advanced imaging methods such as computed tomography/magnetic resonance (CT/MR) are used to diagnose the disease. Treatment usually involves a combination of surgery, chemotherapy, and sometimes radiotherapy and has a high success rate when the disease is diagnosed at an early stage. However, due to the lack of symptoms in the early stages, ~70% of cases are diagnosed in advanced stages, which negatively affects the success of treatment (3). High-grade serous ovarian cancer (HGSOC) is considered the most aggressive subtype of the disease, accounting for 70% of all ovarian cancer cases and approximately 95% of diagnosed cases. This type of cancer is usually diagnosed at an advanced stage, with metastatic spread being common. These factors significantly limit treatment options and make it difficult to determine treatment strategies (4, 5). Standard clinical management of HGSOC involves a treatment paradigm usually characterized by primary reduction surgery (PDS) followed by platinum- and taxane-based chemotherapy (6). Optimal cytoreduction aims to reduce the tumor burden to the level of residual disease (R0), and achieving R0 is known to improve the response to adjuvant chemotherapy significantly. However, the presence of extensive disease in ~70% of stage III and IV ovarian cancer patients complicates optimal resection and requires the consideration of neoadjuvant chemotherapy (NACT) in the majority of cases. In recent years, NACT has emerged as an approach for the management of HGSOC, the most common and aggressive subtype of advanced epithelial ovarian cancer, in combination with interval debulking surgery (IDS), with the potential to achieve complete resection. According to two large prospective randomized trials, R0 rates ranged from 35 to 51% in patients undergoing NACT, whereas they ranged from 15 to 19% in the PDS group. Achieving R0 status has been shown to have a positive effect on clinical outcomes such as progression-free survival and overall survival (7, 8). In this context, the prediction of R0 and partial resection (R1) classification after neoadjuvant treatment plays a critical role in the management of the treatment process.

Proteomic technologies provide a comprehensive analysis of proteins in cells and tissues, allowing the identification of specific proteins involved in the development and progression of cancer. In particular, when methods such as mass spectrometry and microarrays are used, cancer cell-specific proteins can be profiled. In this way, valuable biomarkers are being discovered for diagnosing the early stages of the disease, determining patient prognosis, and monitoring patient response to treatment. Furthermore, proteomic analyses contribute to the development of targeted treatment strategies for individualized medicine practices, enabling more effective management of patients. In this context, proteomics technologies have the potential to revolutionize cancer research and clinical applications (9, 10). In recent years, artificial intelligence (AI) and, in particular, explainable artificial intelligence (XAI) techniques have made significant advances in medical data analysis. These technologies are used to develop predictive models by analyzing large and complex datasets and have great potential to predict treatment responses and support personalized medicine applications. AI-based models offer new approaches to identify biomarkers from proteomic data and improve classification accuracy (11–13).

AI and proteomics technologies play critical roles in identifying biomarkers in diseases such as cancer; proteomics analyses elucidate the mechanisms of disease by studying protein profiles, whereas AI analyses these data to reveal meaningful patterns. This interaction allows for early diagnosis and the development of personalized treatment strategies. The first aim of this study was to predict residual disease status before NACT in HGSOC patients via three different artificial intelligence models on the basis of proteomic data. The second aim is to clinically interpret possible biomarkers using SHapley Additive exPlanations (SHAP), one of the XAI models applied to the optimal model for classification.

## 2 Materials and methods

### 2.1 Dataset

The open-access dataset used in this study consists of proteomic data from 20 patients who were diagnosed with HGSOC and treated with NACT (14). These patients underwent interval debulking surgery (IDS) after NACT treatment and were divided into two groups according to surgical outcomes: no residual disease (R0, *n* = 14) and suboptimal residual disease (R1, *n* = 6). The R0 group consisted of patients with no residual tumor even at the microscopic level after surgery, whereas the R1 group included patients with residual tumor between 0.1 and 1 cm after surgery. Within the scope of the study, tumor tissues were collected from patients both before NACT (pre-NACT) and after NACT (post-NACT) and were isolated using the laser microdissection method, and label-free proteomic analysis was performed. A total of 4,336 proteins were detected, of which 3,043 were quantitatively analyzed. In the analyses performed before and after NACT, 97 proteins were found to be significantly different. In this study, 97 proteins in pre-NACT tissues that were significantly different between R1 and R0 patients (LIMMA *p* < 0.05) were used in machine learning models to predict residual disease (R0, R1) after NACT. This study was approved by the Inonu University Health Sciences Non-Interventional Clinical Research Ethics Committee (approval number: 2024/6557).

### 2.2 Data preprocessing and development of machine learning models

In machine learning models, class imbalance (where one class has far fewer observations than the others) can lead the model to predict the majority class more often, biasing the prediction accuracy and validity. This can cause the model to produce inaccurate results and lead to performance problems, such as low sensitivity, especially when the minority class is important (15). In this study, to address class imbalance (R0 = 14, R1 = 6), we used the ovun.sample function in the R programming language library Random Over-Sampling Examples (ROSE), thus implementing both oversampling and under-sampling (16). In contrast, variable selection methods, which play a critical role in machine learning to improve model performance, reduce the computational burden and improve the generalization capability of the model, ensure that only the most important variables are used in the model by eliminating unnecessary or low-information features in the data. Variable selection prevents the model from overfitting, leading to a more meaningful analysis. Especially in datasets with many variables, variable selection can significantly increase the speed and accuracy of the model (17). In the present study, BORUTA was used as a variable selection method. BORUTA is a variable selection method based on the random forest algorithm. BORUTA evaluates the importance of each variable by creating “shadow features” and comparing them with each other. These shadow variables are randomly shuffled versions of the original variables, and BORUTA tests whether a variable is more important than these random shadows. If a variable is found to be statistically more significant than the shadow variables, it is considered important. This method allows for the evaluation of all potentially important variables, ensuring that only significant variables remain in the model. The advantage of BORUTA is that it provides a clear ranking of the importance of attributes and minimizes information loss by selecting all significant variables in the data (18). In this study, three different machine learning algorithms, random forest, support vector machines and bootstrap aggregation with classification and regression trees, were used to predict pre-NACT residual disease (R0, R1) on the basis of the pre-NACT values of 97 different protein levels of HGSOC patients with statistically significant differences before and after NACT. Machine learning methods, such as support vector machines (SVM), random forest, and bagged classification and regression tree (CART), have been widely used in cancer classification on the basis of proteomic data. These methods have the potential to provide high accuracy in identifying and classifying biomarkers of cancer types. The often high-dimensional, noisy, and complex nature of proteomic data increases the applicability of such methods and plays a critical role in the diagnosis and prognosis of complex diseases such as cancer (19). Support vector machines (SVMs) effectively capture complex patterns via kernel functions for non-linear classification (20). The random forest method offers the ability to generalize by detecting data variations via the combination of multiple decision trees, which provides significant advantages in terms of feature selection and accuracy (21). Bagged CART, in contrast, produces consistent and interpretable classifications via the integration of decision trees with bagging (22). These three methods stand out as effective and reliable tools for cancer classification. To evaluate the performance of the machine learning models, the dataset was divided via stratified random sampling, with 70% allocated for training and 30% allocated for testing. The grid search method, which uses five repeated and 10-fold cross-validations, has been employed to optimize the hyper parameters of machine learning models. The effectiveness of each model was evaluated via a test set, and the results were compared. Among the performance indicators for all the models are accuracy, specificity, sensitivity, area under the receiver operating characteristic (ROC) curve (AUC), F1-score, and Brier score. To comprehensively evaluate the model's performance, we used the Brier score to examine the reliability and calibration of the predictions and the AUC metric to evaluate the ability to separate classes accurately. The machine learning model with the best result according to these two-performance metrics is selected for global explanations with XAI.

### 2.3 Random forest (RF)

The random forest (RF) is a powerful and flexible supervised learning algorithm that is often used in classification and regression problems. It applies the ensembling technique by combining multiple decision trees and training each tree with randomly selected samples (bootstrap samples). A large number of decision trees reduces the risk of overfitting and improves accuracy. Especially in complex datasets, final results based on majority voting or averaging predictions can provide more accurate classification and prediction results than a single decision tree. However, the use of multiple trees increases the computational cost and complicates the interpretability of the model. Parameter optimization (e.g., number of trees and maximum depth) can significantly affect model performance, which can also inform feature selection. The RF algorithm can produce fast and efficient results on large datasets with its parallel processing structure (23).

### 2.4 Support vector machine (SVM)

Support vector machines (SVMs) are powerful supervised learning algorithms used for classification and regression tasks and are particularly effective for high-dimensional datasets. The SVM performs classification by determining an optimal hyperplane that separates the data into two classes. This hyperplane maximizes the margin (the distance to the closest data points) between the classes, reducing the overall error. It also projects the data into a higher-dimensional space via kernel functions for datasets that cannot be linearly separated, thereby achieving non-linear decision boundaries. This flexible structure improves the accuracy of the SVM and minimizes the risk of overfitting. However, with large datasets, the training time can be long, and the optimization process of the model can become complex because of the importance of parametric adjustments (24).

### 2.5 Bootstrap aggregation with classification and regression trees (bagged CART)

Bagged CART (bootstrap aggregation with classification and regression trees) uses the bootstrap aggregation technique to create many independent trees to improve the performance of decision tree algorithms. This method divides the training data into multiple subdatasets via random sampling and provides robustness against data variations by training an independent decision tree in each subdataset. Bagging combines the predictions of each tree model, uses majority voting for classification problems, and averages the predictions for regression problems. This aggregation strategy reduces the risk of overfitting and improves the accuracy of the model. The advantage of bagged CART is that it balances the complexity of the model, resulting in high performance even on large datasets and complex feature spaces. However, it requires training a large number of decision trees, which may increase the computational cost and reduce the interpretability of the model (25).

### 2.6 Explainable artificial intelligence; SHapley Additive exPlanations (SHAP)

Explainable artificial intelligence (XAI) is an approach that aims to make the inner workings of AI models transparent and present how their decisions are made in a manner understandable to humans. This is especially important for algorithms such as deep learning, machine learning, and ensemble models, which are complex and so-called “black boxes”. XAI allows us to understand the reasons for the results of these models and assess the reliability, accuracy, and fairness of the model (26). SHapley additive exPlanations (SHAP) is one of the most popular techniques used for XAI and uses game theory to explain the predictions of a model. SHAP makes the model's decisions more understandable by scoring the contribution of each attribute to the model output as positive or negative. This allows for a more transparent analysis of the extent to which models use which features and how they generate the final predictions (27).

### 2.7 Statistical analysis

The demographic characteristics of the HSGOC patients included in the study are summarized in numbers and percentages. In addition, groupwise descriptors for the 18 proteins considered after BORUTA variable selection are presented as the mean ± standard deviation and median (min–max). The conformity of the 18 proteins included in the model to the normal distribution on a group basis was evaluated via the Shapiro–Wilk test. For proteins that met the assumption of a normal distribution on a group basis, the statistical difference between the groups was evaluated via two-sample *t*-tests, whereas for proteins that did not meet the assumption of a normal distribution on at least one group basis, the statistical difference between the groups was evaluated via the Mann–Whitney U test. *p* < 0.05 was considered statistically significant. Statistical Package for the Social Sciences (SPSS) version 26.0 (28) was used for the statistical analyses, and the R (29) and Python (30) programming languages were used for the development of the machine learning models.

## 3 Results

The clinical data for the 20 HGSOC patients included in the study are depicted in Table 1. In this study, 18 proteins were selected via the BORUTA method among 97 proteins whose protein levels were found to differ between the two groups. The descriptive statistics for the proteins included in the model, as determined by the BORUTA method, are presented in Table 2 for residual disease in HGSOC patients within the scope of this study.

**Table 1:** Clinical data for the 20 HGSOC patients included in the study.

**Clinical characteristic**		**Count (%)**
Age of diagnosis (year)	< 50 years old	4 (20%)
	50–59 years old	9 (45%)
	>60 years old	7 (35%)
Stage	III NOS	3 (15%)
	IIIB	1 (5%)
	IIIC	5 (25%)
	IV NOS	3 (15%)
	IVA	3 (15%)
	IVB	5 (25%)

NOS, Not otherwise specified.

**Table 2:** Descriptive statistics for proteins included in the model after the BORUTA method in terms of residual disease.

**Protein ID**	**Protein name**	**Group**				***p*-value**
		**R0 (*****n*** = **14)**		**R1 (*****n*** = **6)**		
		**Mean ±SD**	**Median (min–max)**	**Mean ±SD**	**Median (min–max)**	
O75874	Isocitrate dehydrogenase [NADP] cytoplasmic	−0.867 ± 0.486	−0.944 (−1.59–0.277)	−0.29 ± 0.559	−0.39 (−0.836–0.627)	**0.032** ^*^
P78417	Glutathione S-transferase omega-1	−0.782 ± 0.758	−0.858 (−1.865–0.569)	−0.006 ± 0.589	0.211 (−1.063–0.575)	**0.032** ^*^
P11766	Alcohol dehydrogenase class-3	−0.998 ± 0.694	−1.094 (−2.554–0.224)	−0.361 ± 1.088	−0.437 (−1.976–1.449)	0.117^*^
P12955	Xaa-Pro dipeptidase	−0.48 ± 0.469	−0.386 (−1.316–0.137)	−0.082 ± 0.257	−0.114 (−0.408–0.356)	0.083^*^
P07305	Histone H1.0	−0.764 ± 0.711	−0.887 (−1.871–0.31)	−0.393 ± 0.715	−0.655 (−0.829–1.032)	0.409^**^
Q14914	Prostaglandin reductase 1	−0.16 ± 0.593	−0.217 (−1.258–1.26)	−0.634 ± 0.672	−0.734 (−1.298–0.392)	0.248^*^
P00568	Adenylate kinase isoenzyme 1	−0.438 ± 0.493	−0.359 (−1.15–0.329)	0.149 ± 0.453	0.252 (−0.597–0.717)	**0.021** ^*^
P49189	4-trimethylaminobutyraldehyde dehydrogenase	−0.54 ± 0.485	−0.537 (−1.159–0.668)	−0.053 ± 0.408	−0.125 (−0.378–0.736)	**0.017** ^**^
P48637	Glutathione synthetase	−0.56 ± 0.654	−0.47 (−1.97–0.435)	0.093 ± 0.407	0.061 (−0.42–0.81)	**0.026** ^*^
Q8NCW5	NAD(P)H-hydrate epimerase	−0.702 ± 0.419	−0.801 (−1.449–0.045)	−0.284 ± 0.51	−0.225 (−1.063–0.436)	0.099^*^
P48739	Phosphatidylinositol transfer protein beta isoform	−0.085 ± 0.601	−0.108 (−0.892–1.211)	−0.187 ± 0.164	−0.212 (−0.387–0.014)	0.741^*^
O43491	Band 4.1-like protein 2	−0.557 ± 0.516	−0.549 (−1.456–0.368)	0.079 ± 0.574	−0.042 (−0.513–0.985)	**0.021** ^*^
P35237	Serpin B6	−0.352 ± 0.341	−0.354 (−0.952–0.197)	0.056 ± 0.468	−0.061 (−0.411–0.937)	0.070^*^
Q96CX2	BTB/POZ domain-containing protein KCTD12	−0.298 ± 0.34	−0.353 (−0.794–0.345)	0.001 ± 0.243	−0.041 (−0.272–0.321)	**0.032** ^**^
P25786	Proteasome subunit alpha type-1	−0.341 ± 0.294	−0.293 (−0.815–0.06)	−0.061 ± 0.196	−0.063 (−0.368–0.172)	**0.048** ^*^
Q96T58	Msx2-interacting protein	0.355 ± 0.189	0.351 (0.016–0.631)	0.111 ± 0.292	0.084 (−0.27–0.525)	0.070^*^
O95302	Peptidyl-prolyl cis-trans isomerase FKBP9	−0.67 ± 0.75	−0.81 (−1.75–0.77)	−0.09 ± 0.30	−0.03 (−0.56–0.29)	**0.041** ^*^
P02788	Lactotransferrin	−0.803 ± 1.563	−1.029 (−2.423–3.927)	−0.497 ± 0.642	−0.489 (−1.428–0.544)	0.117^**^

SD, standard deviation; Min, minimum; Max, maximum; ^*^Independent sample *t*-test; ^**^Mann–Whitney U test. Bold values indicate statistical significance (*p* < 0.05) between R0 and R1 groups.

Considering the statistics given in Table 2, the regulation of nine proteins (O75874, P78417, P00568, P49189, P48637, O43491, Q96CX2, P25786, and O95302) significantly differed between the two groups (R0 and R1) (*p* < 0.05). Table 3 shows the performance metrics (AUC, accuracy, sensitivity, specificity, F1-score, and Brier score) for three different machine learning methods (RF, SVM, and Bagged CART) on the basis of the data obtained after proteomic analyses were performed on formalin-fixed, paraffin-embedded (FFPE) tumor tissues from HGSOC patients before NACT to predict residual disease status (R0, R1) before NACT.

**Table 3:** The performance metrics (AUC, accuracy, sensitivity, specificity, F1-score, and Brier score) of the RF, SVM, and Bagged CART models for predicting residual disease status (R0, R1) from pre-NACT data.

**Model**	**AUC**	**Accuracy**	**Sensitivity**	**Specificity**	**Brier score**	**F1-score**
Bagged CART	91.0	78.7	89.6	67.3	0.146	79.9
Random Forest	95.5	83.0	90.4	76.3	0.105	83.9
SVM	93.0	85.2	89.5	83.1	0.113	84.5

AUC, area under the receiver operating characteristic (ROC) curve.

**Figure 1:**
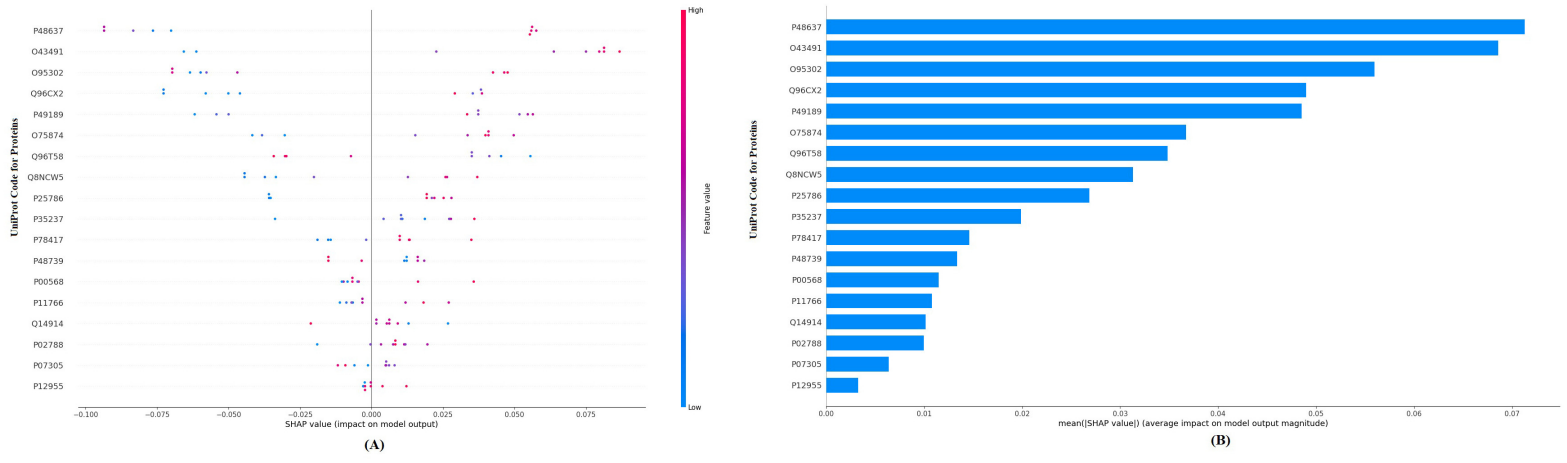
**(A)** Global SHAP annotations of the random forest model for residual disease prediction. The bee swarm plot shows how features in the model affect predictions. Each dot represents a data sample, and the positions of the dots on the *x*-axis represent SHAP values (positive or negative impact). The colors of the dots represent feature values (blue—low, red—high). **(B)** Protein importance plots based on the mean SHAP values of the random forest model for residual disease prediction. The bar graph shows the average of the absolute SHAP values of the marginal contribution of each variable to the model output. This graph presents the relative importance of the variables on the model predictions in a hierarchical structure.

When considering the performance metrics for the three different machine learning methods, the best classification performance is the random forest model, with an AUC of 95.5% and a Brier score of 0.105. Figure 1A visualizes the SHAP values for the residual disease optimal model random forest with a bee swarm plot, which is used for visualizing data points for global interpretation in explainable artificial intelligence (XAI) applications. The bee swarm plots illustrate the importance of the predictors included in the model in classification as well as their positive/negative associations with the target variable. Each point in the graph represents a sample in the data, while the colors indicate the relative values of the variables. For residual disease prediction, blue and red indicate low and high values for biomarker candidate proteins, respectively. Thus, low values of the accession-encoded proteins P48637, O43491, O95302, Q96CX2, P49189, and O75874 and high values of the accession-encoded proteins Q96T58 and P48739 increase the risk of no residual disease after NACT. Figure 1B shows the protein importance plots of the most critical proteins in descending order for the optimal random forest model in the tumor size prediction task on the basis of the aggregated SHAP values. The length of each bar represents the average of the absolute SHAP values for the protein(s) of interest. According to Figure 1B, the top five most important proteins for predicting residual disease after NACT are those with access codes P48637, O43491, O95302, Q96CX2, and P49189, respectively.

## 4 Discussion

In HGSOC, the most common and deadliest subtype of epithelial ovarian carcinoma, preoperative prediction of resection status before neoadjuvant chemotherapy (NACT) and optimization of early diagnosis/treatment strategies have multifaceted importance in the clinical management of the disease. This is particularly evident in clinical situations where 5-year survival rates decline from 70 to 90% in early-stage tumors to 25–30% in advanced-stage tumors, and optimal cytoreductive surgery is one of the major prognostic factors affecting long-term prognosis. The estimation of residual disease before NACT provides important benefits for the optimization of treatment strategies, such as the choice of primary surgery or NACT, assessing complication risks, and planning postoperative care. This approach improves patient quality of life with less invasive surgical procedures and shorter hospital stays and has a positive impact on the healthcare system in terms of resource efficiency. Recent studies have identified various mutations in ovarian cancer, highlighting the role of tumor biomarkers in diagnosis and treatment. In particular, BRCA1/2 mutations and homologous recombination repair deficiency (HRD) have been shown to significantly influence the chemotherapy response. The identification of these biomarkers allows for the development of personalized therapeutic strategies, including the use of PARP inhibitors, which have shown high antitumor activity in BRCA-mutated and HRD-positive patients (31). These inhibitors are currently being evaluated in clinical trials for their antitumor potential. Notably, mutations in the RAS-RAF-MEK-ERK pathway have been implicated in resistance to conventional chemotherapy, particularly in low-grade serous ovarian cancer (LGSOC). Compared to standard therapies, v-raf murine sarcoma viral oncogene homolog B1 (BRAF) and mitogen-activated protein kinase (MEK) inhibitors, which are designed to target this pathway, have shown promising antitumor activity, especially in patients with the BRAF V600E mutation, leading to higher response rates. These findings highlight the importance of molecular profiling in predicting treatment response and optimizing therapeutic strategies (32).

In addition, providing scientific contributions, such as the development of predictive models based on artificial intelligence, the identification of risk factors, and the standardization of treatment protocols, contributes significantly to both improving individual patient outcomes and increasing the efficiency of the healthcare system. In a study aimed at developing a machine learning-based immune risk model (immune-risk tumor microenvironment (TMErisk) model) that can predict prognosis and identify treatment strategies for HGSOC, the authors defined two different immune microenvironment phenotypes on the basis of immune and stromal cell signatures and developed a clinically applicable prognostic scoring system using 10 independent machine learning algorithms. The low-TMErisk group was characterized by BRCA1 mutation, immune activation, and a better immune response, whereas the high-TMErisk group was associated with the deletion of C-X-C motif chemokine ligands and carcinogenic activation pathways. TMErisk outperforms other clinical characteristics and published signatures, and patients in the low-TMErisk group were observed to respond better to immunotherapy and chemotherapy. This study may contribute to the development of more personalized and effective approaches for ovarian cancer treatment (33). In another study, machine learning models were developed to predict sensitivity to platinum-based therapy in HGSOC. The researchers analyzed clinicopathological data from a total of 1,002 HGSOC patients from three different hospitals and identified six variables (age, baseline serum CA-125 levels, neoadjuvant chemotherapy, pelvic lymph node status, pelvic tissue involvement other than the uterus and tubes, and small bowel and mesentery involvement) associated with platinum sensitivity via a stepwise selection method. On the basis of these variables, prediction models were developed via four different machine learning algorithms (logistic regression, random forest, support vector machine, and deep neural network). The logistic regression-based model performed best in identifying platinum-resistant cases, with an AUC of 0.741. A web-based nomogram was also developed for clinical use, providing a tool to help implement individualized treatment and follow-up protocols (34).

Differing from the TMErisk model and platinum sensitivity prediction studies, this study used pre-NACT proteomic data from 20 patients diagnosed with HGSOC, and residual disease (R0, R1) was classified using three different machine learning methods (BaggedCART, Random Forest, and SVM) created on the basis of these data. When all the performance metrics were considered, the random forest model was the best prediction model, with AUC, accuracy, sensitivity, specificity, Brier score, and F1-score values of 0.955, 0.830, 0.904, 0.763, 0.105, and 0.839, respectively. These results show that the random forest model performs exceptionally well in predicting residual disease status in HGSOC patients. In particular, the AUC value of 0.955 indicates that the model has a very high ability to distinguish classes. The accuracy rate of 0.830 and the F1-score value of 0.839 indicate that the overall prediction performance of the model is consistent and balanced. The sensitivity value of 0.904 indicates that the model is quite successful in correctly detecting positive cases (R1). In contrast, the specificity value of 0.763 indicates that it can discriminate negative cases (R0) at a satisfactory level. A low Brier score value of 0.105 indicates that the predictions of the model are reliable and calibrated. These results suggest that pre-NACT proteomic data can be valuable biomarkers for the prediction of residual disease status and that the random forest algorithm is an effective tool for such clinical prediction tasks. The five proteins most important for predicting residual disease after NACT with SHAP analysis via XAI methods applied to the optimal random forest model are those with accession codes P48637 (glutathione synthetase), O43491 (band 4.1-like protein 2), O95302 (peptidyl-prolyl cis-trans isomerase FKBP9), Q96CX2 [Broad-Complex, Tramtrack, and Bric-a-brac/POxvirus and Zinc finger (BTB/POZ)] domain-containing protein (potassium channel tetramerization domain-containing protein 12 [KCTD12]), and P49189 (4-trimethylaminobutyraldehyde dehydrogenase). Moreover, according to the statistical analysis, these five key proteins, which were significantly different between the two groups, stand out as important biomarkers in the prediction of residual disease after NACT. The importance ranking of these proteins quantitatively reveals the effects of the model in the decision-making process. These proteins, which are determined with SHAP values, both increase the interpretability of the model and can guide the determination of potential therapeutic targets. In addition, the high predictive power of these proteins can contribute to a better understanding of the molecular basis of the NACT response in HGSOC and can be used to optimize treatment strategies. These findings constitute an important step in the development of personalized treatment approaches and more accurate assessments of patient prognosis.

While this study focused on predicting residual disease in HGSOC via proteomic biomarkers, integrating molecular profiling with predictive models could further enhance treatment strategies. In particular, the inclusion of BRCA mutations and HRD status could improve the model's ability to predict chemotherapy response, enabling more personalized and effective therapeutic interventions. The success of the use of BRAF and MEK inhibitors in LGSOC emphasizes the potential of personalized medical approaches that target molecular alterations. These findings suggest that the integration of molecular profiling with predictive models could improve treatment strategies for different ovarian cancer subtypes. Furthermore, the development of advanced molecular profiling techniques could improve the performance of AI-based prediction models by identifying subtype-specific biomarkers.

Future research should explore the combined use of AI-based residual disease prediction models and molecular profiling, including tumor biomarkers such as BRCA mutations and HRD status. This integrative approach could lead to more personalized and effective treatment strategies for ovarian cancer, optimizing therapeutic outcomes by accurately predicting the chemotherapy response. Additionally, understanding the molecular differences between HGSOC and LGSOC remains crucial for tailoring treatment approaches and enhancing patient-specific care. Such integrative approaches may lead to more personalized and effective treatment strategies for ovarian cancer. In this context, understanding the molecular differences between HGSOC and LGSOC will be crucial for developing tailored therapeutic interventions.

Glutathione (GSH) serves as a critical cellular antioxidant and reducing agent, playing multifaceted roles in various physiological and cellular processes. It is integral to the metabolism of xenobiotics and cellular molecules, scavenges free radicals, regulates cell cycle dynamics, and maintains microtubule integrity. GSH also functions as a physiological reservoir of cysteine (Cys), modulates calcium (Ca^2+^) homeostasis, and regulates protein function and gene expression through thiol–disulfide exchange reactions. Furthermore, it contributes to immune modulation, lymphocyte function, and mitochondrial mechanisms linking permeability transition pore complexes to the activation of cell death. In addition to these roles, GSH plays a pivotal role in maintaining the intracellular redox balance and is heavily implicated in cellular processes such as differentiation, proliferation, and apoptosis. Additionally, it has been associated with resistance to ionizing radiation and drug-induced cytotoxicity. In the present study, the mean level of the protein encoded by P48637 (glutathione synthetase) was significantly greater in the partial resection group following neoadjuvant chemotherapy. Cisplatin and carboplatin, two platinum-based chemotherapeutic agents, have similar efficacy profiles, with carboplatin exhibiting reduced toxicity compared with cisplatin. Importantly, GSH mediates resistance to these agents through several mechanisms, including reduced drug uptake, enhanced intracellular detoxification/inactivation of the drug, improved DNA repair, and inhibition of apoptosis triggered by drug-induced oxidative stress (35). In ovarian cancer, high levels of GSH and glutathione S-transferase P1 (GSTP1) activity have been associated with resistance to cisplatin and carboplatin, although some conflicting reports exist (36). Sawers et al. demonstrated that stable deletion of GSTP1 significantly increased the sensitivity of A2780 ovarian cancer cells to both cisplatin and carboplatin (37). Similarly, Crawford and Weerapana identified a dichlorotriazine-containing compound (LAS17) that irreversibly inhibited GSTP1 activity, representing a promising therapeutic target (38). These findings emphasize the complexity of ovarian cancer, particularly concerning the unique metabolic and thiol-related responses to neoadjuvant chemotherapy. The interplay between GSH metabolism and chemotherapeutic efficacy highlights the need to consider these factors in clinical decision-making. Tailoring treatment strategies to account for thiol metabolism and associated resistance mechanisms may improve therapeutic outcomes in ovarian cancer patients.

Band 4.1-like protein 2 (EPB41L2), also known as erythrocyte membrane protein band 4.1-like 2, plays a vital role in mediating the recruitment of the dynein–dynactin complex and NUMA1 to the mitotic cell cortex during anaphase. In this study, the protein associated with O43491 (Band 4.1-like protein 2) was found to have significantly higher levels in the partial resection group following neoadjuvant chemotherapy, suggesting its potential involvement in chemoresistance. Similarly, research by Menyhárt et al. revealed that EPB41L2, one of four genes (alongside HLA-DQB1, LTF, and SFRP1), is consistently overexpressed in tumor samples from ovarian cancer patients with disease progression after topotecan therapy (39). This association supports the notion that EPB41L2 may act as a marker for resistance. The EPB41L2 gene encodes the protein 4.1G, a member of the 4.1 superfamily of scaffold proteins. Unlike its paralogues, protein 4.1G has been linked to poorer survival outcomes, as supported by findings in this dataset and corroborative data from the Human Protein Atlas (40). These observations suggest that EPB41L2 may function as an oncogene in ovarian cancer, although its underlying mechanisms require further investigation. This research underscores the potential of EPB41L2 as both a prognostic biomarker and a therapeutic target, paving the way for precision treatments aimed at overcoming chemoresistance in ovarian cancer.

Peptidyl-prolyl cis-trans isomerase FKBP9, commonly referred to as FK506 binding protein 5 (FKBP5), belongs to the immunophilin family and is defined by its peptidylprolyl cis/trans isomerase (PPIase) activity (41). FKBP5 is a well-recognized target of immunosuppressive agents such as rapamycin and tacrolimus (FK506) and interacts with key proteins, such as Akt and the progesterone receptor (PR), via its FKBP-type domains. This protein plays a central role in several critical signaling pathways, including hormone signaling, NF-κB activation in response to irradiation, and Akt-PHLPP signaling in the context of chemotherapy, emphasizing its multifaceted involvement in cancer progression and resistance to treatment (42). While it shares fundamental characteristics with other FK506-binding proteins, FKBP5 exhibits distinct properties, especially its ability to modulate crucial signaling pathways, such as those driven by Akt (43).

Prior studies have shown that FKBP5 is highly expressed across multiple tissues and significantly contributes to drug resistance in various cancers, including breast and prostate cancers, multiple myeloma, acute lymphoblastic leukemia, and melanoma (44). This resistance often occurs following exposure to antineoplastic therapies such as FK506, rapamycin, dexamethasone, or irradiation. Notably, the present study revealed substantial upregulation of FKBP5 in residual high-grade serous ovarian cancer (HGSOC) tissues from patients undergoing neoadjuvant chemotherapy. These findings further implicate FKBP5 in facilitating chemoresistance in ovarian cancer. These results align with prior research by Sun et al., who examined Taxol-resistant ovarian carcinoma cells derived from the SKOV3 cell line. Their analysis demonstrated that these cells showed cross-resistance to other mitotoxins, such as vincristine, but remained sensitive to the genotoxin cisplatin. Transcriptomic profiling of the Taxol-resistant cells revealed 112 genes with pronounced overexpression, among which FKBP5 exhibited an initial 100-fold upregulation during resistance acquisition, followed by a decline with extended culture. Functional experiments by the same group revealed that silencing FKBP5 resensitized Taxol-resistant cells to Taxol, whereas ectopic overexpression of FKBP5 amplified resistance. This phenomenon was similarly observed with vincristine but not with cisplatin, suggesting the specificity of the role of FKBP5 in mitotoxin resistance (45). These findings suggest a mechanism of FKBP5-mediated chemoresistance that involves intricate protein–protein interactions and transcriptional regulation, suggesting promising therapeutic opportunities for addressing drug resistance in ovarian cancer.

Q96CX2 (BTB/POZ domain-containing protein KCTD12), also known as Pfetin, serves as an auxiliary subunit of gamma-aminobutyric acid (B) (GABAB) receptors and modulates their biophysical and pharmacological properties (46). By increasing agonist potency, accelerating the onset of the receptor response, and promoting desensitization, Pfetin plays a key role in determining receptor pharmacology and the kinetics of G protein signaling (47). Despite its established role in receptor modulation, the involvement of Pfetin in tumorigenesis and cancer progression remains largely unclear. Altered expression of Pfetin has been observed in gastrointestinal stromal tumors (GISTs) with poor clinical outcomes, but the underlying mechanisms regulating its expression are not fully understood (48). In our study, significant upregulation of Pfetin (KCTD12) was detected in residual tissue from patients with high-grade serous ovarian cancer (HGSOC) who had received neoadjuvant chemotherapy, suggesting a potential role for Pfetin in chemoresistance or tumor aggressiveness. Although few studies of Pfetin in ovarian cancer exist, a genome-wide mutation screen identified a mutation in KCTD12 in one patient with high-grade serous ovarian cancer, suggesting its possible involvement in disease progression. Further evidence comes from Suehara et al., who identified Pfetin as a prognostic biomarker in patients with GIST via a proteomic approach. They demonstrated that eight of the identified protein spots were derived from Pfetin, four of which showed high discriminatory power between GISTs with good and poor prognoses. Immunohistochemical analysis of 210 GIST cases confirmed the prognostic value of Pfetin. It revealed that Pfetin-positive tumors were associated with a significantly greater 5-year metastasis-free survival rate (93.9%) than were Pfetin-negative tumors (36.2%). Multivariate analyses also confirmed that Pfetin expression is a decisive prognostic factor independent of clinicopathologic variables, including c-kit or platelet-derived growth factor receptor A mutations (49). Given the similarities between GIST and epithelial ovarian cancers in the acquisition of aggressive features such as invasion, metastasis, and peritoneal dissemination, it is plausible that KCTD12 mutations could lead to a loss of the tumor suppressive function of Pfetin. This loss could drive the acquisition of aggressive phenotypes in ovarian cancer, similar to mechanisms observed in other tumor suppressor genes. These results suggest that Pfetin not only has prognostic value but also may represent a potential therapeutic target. Further studies are needed to clarify the role of KCTD12 and its mutations in the pathophysiology, chemoresistance, and progression of ovarian cancer.

Ovarian cancer is a heterogeneous disease characterized by different histological subtypes and stages of development, which complicates its diagnosis and treatment. The cancer stem cell hypothesis emphasizes the presence of proliferating cell populations in tumors that are capable of self-renewal and differentiation into multiple developmental stages, and contribute to tumorigenesis and progression (50). Among the markers associated with these stem-like cells, aldehyde dehydrogenase (ALDH) proteins have gained attention. ALDH represents a superfamily of 19 enzymes that protect cells from cytotoxic and carcinogenic aldehydes and are distributed across various organelles, including the nucleus, cytosol, mitochondria, and endoplasmic reticulum (51). In addition to their protective function, ALDH enzymes are critical for the maintenance of epithelial homeostasis and have been shown to be important markers of stem cells in both normal and tumor contexts.

Deregulation of ALDH enzymes has been demonstrated in several cancer types, including breast, prostate, lung, and colorectal cancer, and their expression correlates with clinical outcome (52). Additionally, meta-analyses have shown that increased ALDH1 expression in ovarian cancer is associated with poor prognosis, shorter progression-free survival, and unfavorable clinical features (53). Conversely, ALDH5A1 transcription and expression are associated with better overall survival in patients with serous ovarian cancer harboring TP53 mutations but not in patients with wild-type TP53, underscoring the differential role of ALDH enzymes in ovarian cancer pathogenesis and progression (54). In this study, we observed increased levels of P49189, also known as aldehyde dehydrogenase family 9 member A1 (ALDH9A1) or 4-trimethylaminobutyraldehyde dehydrogenase, in residual tissue from patients with high-grade serous ovarian cancer (HGSOC) treated with neoadjuvant chemotherapy. To the best of our knowledge, this is the first report to identify the upregulation of ALDH9A1 in this context, highlighting its potential role in the biology of residual disease and chemoresistance. Using immunohistochemistry, Saw et al. demonstrated significant overexpression of ALDH1A3, ALDH3A2, and ALDH7A1 in ovarian tumors compared with normal ovarian tissue. They also reported tumor type-dependent induction of ALDH enzymes in ovarian cancer cells cultured as sphere suspensions in serum-free medium, suggesting that ALDH expression and activity may vary depending on the cell status and tumor microenvironment. These results indicate that ALDH enzymes play a cell-type-specific role in ovarian tumor tissues and may contribute to stem-like properties and chemoresistance (55). Our results and those of previous studies suggest that elucidating the role of ALDH isozymes, including ALDH9A1, in cell lineage differentiation and tumor progression may provide new insights into the pathophysiology of ovarian cancer. Further studies are needed to understand the regulatory mechanisms of ALDH9A1 and its potential as a prognostic biomarker or therapeutic target in ovarian cancer, particularly in the context of residual disease and chemoresistance.

## 5 Conclusion

This study highlights the effectiveness of machine learning models, particularly random forests, in predicting residual disease status (R0, R1) in high-grade serous ovarian cancer (HGSOC) patients undergoing neoadjuvant chemotherapy (NACT) via proteomic data. The random forest model achieved high predictive accuracy, with an AUC of 95.5% and reliable performance metrics. Key proteins identified through SHAP analysis—such as glutathione synthetase (P48637) and peptidyl-prolyl cis-trans isomerase FKBP9 (O95302)—were found to play significant roles in chemotherapy resistance, suggesting potential targets for personalized therapeutic strategies. This study also demonstrates the power of explainable artificial intelligence (XAI) in enhancing clinical decision-making by making machine learning models more transparent and interpretable. In conclusion, the findings of this research pave the way for integrating machine learning and proteomics to predict treatment outcomes in patients with ovarian cancer, suggesting a promising approach for more individualized and effective therapies. Further research with larger datasets could help validate these biomarkers and optimize treatment strategies for improved patient outcomes.

## Data Availability

The original contributions presented in the study are included in the article/Supplementary material, further inquiries can be directed to the corresponding authors.
