# Supplementary material for: Proteomic alterations in ovarian cancer—Predicting residual disease status using artificial intelligence and SHAP-based biomarker interpretation
Source: Front Med (Lausanne). 2025 Jul 23;12:1562558. doi: 10.3389/fmed.2025.1562558 (PMC12325313; doi:10.3389/fmed.2025.1562558)
Supplement: Supplementary file 1 [file Table_1.docx]

Supplementary Material

# Dataset

**Supplementary Table 1: Proteomic alterations associated with residual disease in neoadjuvant chemotherapy treated ovarian cancer tissues**

| Proteins | R0 | R1 | R1 | R0 | R0 | R1 | R0 | R0 | R0 | R0 | R1 | R0 | R0 | R0 | R0 | R0 | R0 | R1 | R1 | R0 |
| --- | --- | --- | --- | --- | --- | --- | --- | --- | --- | --- | --- | --- | --- | --- | --- | --- | --- | --- | --- | --- |
| Q9NRX4 | 0,158 | 0,669 | -1,277 | 0,874 | -2,280 | -0,448 | -2,167 | -1,705 | -0,654 | -1,645 | -0,405 | 0,764 | -1,516 | -1,009 | -2,229 | -1,024 | -1,603 | -1,663 | -1,132 | 0,314 |
| Q15274 | -2,228 | 0,490 | -0,855 | -0,158 | -2,676 | -2,405 | -1,242 | -0,487 | -0,191 | -0,345 | -0,081 | -0,225 | -1,191 | -1,894 | -1,213 | -1,164 | -1,837 | -1,071 | -1,116 | -1,370 |
| P15121 | -0,523 | 0,627 | -0,732 | -0,211 | -2,819 | -0,148 | -0,771 | -1,070 | -0,371 | -0,484 | -0,718 | 0,269 | -0,718 | -1,022 | -1,791 | -1,716 | -2,509 | -0,387 | -0,378 | 0,127 |
| P51649 | -0,751 | -0,731 | -0,143 | -0,721 | -2,792 | -1,776 | -0,816 | -0,367 | -1,529 | -0,403 | -0,997 | -1,013 | -1,109 | -1,481 | -1,699 | 0,681 | -1,598 | -2,094 | -0,406 | -2,005 |
| O94875 | -1,173 | 0,025 | -1,421 | 0,325 | -1,040 | -0,282 | -0,512 | -0,979 | -1,051 | -0,569 | -0,684 | 0,029 | -1,097 | -1,009 | -1,852 | -0,752 | -0,850 | -1,095 | -0,695 | 0,898 |
| P02768 | -1,794 | 1,129 | -1,446 | -1,172 | -3,171 | -1,974 | -1,633 | -1,140 | -0,346 | 0,420 | -0,373 | -1,446 | -0,794 | -2,175 | -1,716 | -1,694 | -1,361 | -1,254 | 2,043 | -1,699 |
| Q13938 | -1,730 | -0,563 | -0,257 | 0,154 | -1,963 | -0,233 | -0,225 | 0,406 | 0,370 | -0,251 | -0,376 | -0,645 | -0,516 | -1,545 | -1,434 | -1,259 | -1,337 | -1,482 | -0,415 | -1,283 |
| O75874 | -0,988 | 0,627 | -0,836 | -0,637 | -1,590 | -0,295 | -0,668 | -0,306 | -1,200 | -0,560 | -0,806 | -0,802 | -0,905 | -0,983 | -1,096 | 0,277 | -1,559 | 0,055 | -0,485 | -1,117 |
| Q06323 | -0,339 | 0,890 | -0,771 | -0,166 | -1,876 | -0,082 | -1,643 | 0,134 | -0,213 | -0,025 | -0,434 | -1,173 | -1,276 | -1,898 | -1,743 | -0,507 | -1,673 | -2,233 | -0,493 | 0,017 |
| P30044 | 0,098 | -0,564 | -0,385 | 0,757 | -1,832 | -0,836 | -0,584 | -0,261 | -0,352 | -0,450 | -0,208 | -0,528 | 0,567 | -1,554 | -0,804 | -0,247 | -0,634 | -0,622 | -0,161 | -0,825 |
| P78417 | -0,601 | 0,128 | 0,575 | 0,240 | -1,865 | -1,063 | -1,387 | -0,768 | -0,327 | -1,102 | -0,276 | -0,406 | 0,178 | -0,947 | -1,447 | -1,666 | -1,425 | 0,307 | 0,293 | 0,569 |
| P11766 | -1,386 | 1,449 | -0,264 | -0,498 | -1,208 | -1,976 | -1,172 | -1,015 | -1,192 | -0,813 | -0,441 | -0,570 | -0,180 | -0,475 | -1,686 | 0,224 | -2,554 | -0,499 | -0,434 | -1,452 |
| P50135 | -1,085 | 0,695 | -1,035 | -0,335 | -1,295 | -0,728 | -1,371 | -1,669 | -0,748 | -0,977 | -1,531 | -0,044 | -0,615 | -0,763 | -0,883 | 0,030 | -1,017 | -0,689 | -0,886 | 0,147 |
| Q9UQC2 | -0,379 | 0,128 | 0,185 | 0,041 | -1,965 | -1,598 | -0,994 | -0,016 | 0,028 | 0,354 | 0,284 | 0,171 | 0,197 | -1,363 | -1,473 | -0,595 | -1,443 | -0,960 | -1,043 | -1,254 |
| P12955 | -0,064 | -0,015 | -0,199 | -0,148 | -1,089 | -0,408 | -0,176 | -0,331 | 0,038 | -0,323 | 0,356 | 0,137 | -0,645 | -0,612 | -1,300 | -0,442 | -1,316 | -0,194 | -0,033 | -0,444 |
| Q9H1E3 | -1,012 | 0,322 | -0,272 | 0,236 | -0,876 | -0,036 | -0,471 | -1,868 | -0,029 | -0,917 | -0,369 | -0,359 | -1,654 | 0,097 | -0,746 | -0,914 | -1,024 | -1,964 | -0,913 | -0,633 |
| P07741 | -0,851 | 0,628 | 0,221 | 0,510 | -2,852 | -1,728 | -0,567 | -0,472 | -0,119 | 0,107 | 0,189 | -0,448 | -0,206 | -1,343 | -0,770 | 0,184 | -1,191 | -0,485 | 0,179 | -0,569 |
| Q9NQR4 | 0,085 | -0,639 | -0,753 | -0,013 | -1,936 | 0,206 | -0,447 | 0,047 | -0,914 | -1,083 | -1,211 | -0,286 | -0,485 | -1,355 | -1,641 | -0,515 | -1,631 | -0,924 | -0,220 | -0,582 |
| P17174 | -1,252 | -0,593 | -0,164 | 0,094 | -2,024 | -1,275 | -1,010 | -0,201 | 0,230 | -0,718 | 0,238 | 0,396 | -0,319 | -1,248 | -1,540 | 0,027 | -1,542 | -0,582 | -0,160 | -0,266 |
| Q9UBQ7 | -1,268 | 0,576 | -0,519 | 0,115 | -1,366 | -0,019 | -0,778 | -0,232 | -0,619 | -0,177 | -0,545 | -0,240 | -0,254 | -0,927 | -0,366 | 0,199 | -0,219 | -1,210 | -0,326 | -0,409 |
| P14174 | -0,746 | -0,306 | -1,090 | 0,175 | -0,483 | -0,305 | -0,602 | -1,384 | -0,091 | -1,937 | -0,927 | 0,084 | -1,553 | -0,551 | -1,338 | -0,634 | -1,217 | -0,842 | -0,276 | -0,583 |
| Q08257 | -0,795 | 0,510 | -0,552 | -0,473 | -0,839 | 0,621 | -0,985 | -0,802 | -0,926 | -0,475 | -0,827 | -0,652 | 0,024 | -0,297 | 0,014 | 0,167 | 0,065 | -0,295 | -0,266 | 0,039 |
| P07108 | -1,118 | 0,216 | -0,705 | 0,121 | -1,939 | -0,173 | -0,440 | -0,488 | 0,310 | -0,330 | 0,130 | -0,112 | -1,184 | -1,252 | -1,791 | -0,454 | -1,959 | -0,572 | -0,138 | -0,055 |
| P07305 | 0,310 | 1,032 | -0,450 | -0,249 | -1,634 | -0,829 | -0,265 | -1,170 | -0,741 | -0,484 | -0,534 | 0,159 | -1,033 | -1,302 | -1,871 | -1,176 | 0,169 | -0,799 | -0,777 | -1,411 |
| Q14914 | 0,444 | 0,392 | -1,298 | 1,260 | -0,340 | -0,268 | -1,258 | -0,400 | -0,022 | 0,213 | -1,141 | -0,094 | -0,811 | 0,075 | -0,430 | -0,021 | -0,510 | -0,326 | -1,163 | -0,345 |
| P00568 | 0,329 | 0,207 | 0,717 | 0,047 | -1,031 | -0,597 | -0,907 | -0,696 | -0,336 | -0,136 | -0,109 | 0,098 | -0,381 | -0,777 | -1,150 | 0,087 | -0,236 | 0,298 | 0,382 | -1,047 |
| Q8N335 | 0,689 | -0,055 | -0,326 | 0,504 | -1,993 | -0,467 | -0,647 | -0,096 | -0,014 | 0,077 | -0,672 | -0,074 | 0,496 | -1,715 | -0,528 | 0,505 | -0,051 | -0,620 | 0,465 | -0,147 |
| P40925 | -0,741 | 0,730 | -0,321 | 0,411 | -1,679 | -0,640 | -1,308 | -0,644 | -0,273 | -0,932 | -0,715 | -0,277 | -0,600 | -1,086 | -0,678 | 0,033 | -0,880 | -0,444 | -0,169 | -0,588 |
| P00441 | -0,251 | 0,469 | 1,304 | 0,519 | -0,806 | -1,121 | -1,443 | -0,650 | -0,671 | -1,177 | 0,032 | -0,117 | -0,524 | -0,981 | -1,000 | -0,820 | -0,836 | -0,380 | -0,598 | -0,829 |
| P60174 | 0,044 | 0,240 | -0,939 | 0,257 | -1,265 | -0,183 | -1,133 | -0,683 | -0,360 | -0,681 | -0,203 | 0,395 | -0,349 | -0,538 | -1,407 | -0,852 | -1,792 | -0,717 | -0,435 | -0,424 |
| O95302 | -0,319 | -0,563 | -0,077 | -0,143 | -1,292 | 0,292 | -0,466 | -1,362 | 0,630 | -1,410 | 0,049 | 0,769 | -0,335 | -1,168 | -1,747 | -0,797 | -0,963 | -0,281 | 0,014 | -0,828 |
| O15230 | -0,662 | 0,509 | -0,368 | 0,165 | -0,188 | 0,273 | -0,409 | -0,002 | -0,347 | -0,206 | -0,363 | -0,799 | -0,262 | -0,908 | -1,170 | -0,654 | -1,137 | -0,629 | -0,422 | -0,407 |
| P30533 | -0,050 | -0,140 | -0,426 | -0,584 | -0,264 | 0,091 | -0,955 | -0,773 | 0,471 | -0,953 | -0,377 | 0,710 | -0,549 | -0,064 | -0,353 | -0,104 | -0,305 | -0,244 | -1,046 | 0,390 |
| Q9Y3D6 | -1,019 | 0,380 | -0,764 | -1,149 | -0,349 | 0,277 | -0,414 | 0,696 | -0,337 | 0,184 | -0,659 | -0,153 | -0,794 | -0,358 | 0,651 | 0,326 | 0,026 | -0,833 | -0,559 | -0,828 |
| P49189 | -0,437 | 0,736 | -0,071 | -0,004 | -1,105 | -0,155 | -0,925 | -1,159 | -0,933 | -0,368 | -0,095 | -0,400 | -0,583 | -0,955 | -0,515 | 0,668 | -0,558 | -0,357 | -0,378 | -0,282 |
| P48637 | -0,668 | 0,135 | 0,140 | -0,181 | -1,970 | 0,810 | -0,603 | -0,717 | 0,095 | -0,219 | -0,014 | 0,435 | -0,337 | -1,146 | -1,455 | 0,118 | -0,914 | -0,420 | -0,095 | -0,275 |
| P07602 | -0,504 | 1,130 | -0,305 | -0,405 | -0,294 | -0,634 | -0,655 | -0,764 | -0,417 | 0,046 | -0,427 | -0,430 | 0,058 | -1,371 | -1,041 | 0,764 | -0,372 | -0,210 | -0,780 | -1,362 |
| Q8NCW5 | -0,976 | -0,023 | -0,605 | 0,045 | -1,449 | 0,436 | -0,772 | -0,835 | -0,501 | -0,274 | -0,226 | -0,208 | -1,185 | -0,884 | -0,726 | -0,227 | -1,001 | -1,063 | -0,225 | -0,830 |
| Q99569 | 0,075 | 0,057 | -0,343 | 0,680 | -0,676 | -0,742 | -0,640 | 0,037 | 0,258 | -0,137 | 0,218 | -0,094 | -0,320 | -0,874 | -1,120 | -1,461 | -1,404 | -1,012 | -0,215 | -0,431 |
| P48739 | 0,406 | -0,136 | -0,288 | -0,222 | -0,673 | -0,387 | -0,156 | 0,516 | 0,043 | -0,059 | 0,014 | 1,211 | -0,892 | -0,445 | -0,739 | 0,407 | -0,787 | -0,021 | -0,306 | 0,201 |
| P14550 | -0,724 | 0,489 | -0,493 | 0,094 | -1,431 | -0,640 | -0,332 | -0,744 | -0,591 | -0,123 | -0,481 | -0,397 | -0,395 | -1,065 | -0,827 | 0,374 | -0,549 | -0,376 | -0,234 | -0,362 |
| O95336 | -0,242 | -0,237 | -0,163 | -0,033 | -1,588 | -0,664 | 0,019 | -0,801 | 0,204 | 0,069 | -0,143 | -0,400 | 0,017 | -0,929 | -0,202 | -0,761 | -0,436 | -0,368 | -0,095 | -0,514 |
| Q9NT62 | -0,432 | 0,321 | -0,039 | 0,149 | -0,460 | -0,874 | -0,087 | -0,451 | -0,201 | -0,471 | -0,944 | 0,132 | -0,175 | -0,319 | -0,429 | -0,181 | -0,427 | -0,370 | 0,053 | -0,778 |
| P29401 | 0,092 | -0,263 | -0,399 | 0,182 | -1,527 | 0,524 | -0,177 | -0,446 | -0,078 | -0,598 | -0,718 | 0,461 | 0,064 | -0,604 | -1,081 | -0,413 | -1,620 | -0,420 | -0,208 | -0,737 |
| P55268 | -0,146 | 0,392 | -0,185 | 0,291 | -0,700 | -0,226 | -0,412 | -0,181 | -0,134 | 0,005 | -0,046 | -0,440 | -0,240 | -0,900 | -0,888 | -0,733 | -0,807 | -0,550 | -0,166 | -0,285 |
| Q99497 | 0,597 | -0,110 | -0,268 | 0,864 | -0,933 | -0,480 | -0,203 | -0,114 | 0,170 | -0,015 | 0,498 | -0,115 | -0,042 | -0,748 | -0,857 | -0,861 | -1,059 | -0,308 | -0,106 | -0,177 |
| Q12765 | -0,034 | 0,172 | -0,652 | -0,023 | -2,006 | -0,844 | -0,347 | -0,938 | 0,474 | -0,940 | 0,817 | 0,826 | 0,003 | -0,436 | -1,116 | -1,028 | -0,813 | -0,505 | -0,756 | -0,636 |
| P22570 | -0,516 | 0,108 | -0,464 | -0,526 | -0,482 | 0,557 | -0,424 | 0,194 | -0,177 | -0,249 | -0,084 | -0,591 | -0,143 | -0,530 | -0,345 | -0,121 | 0,057 | 0,169 | -0,527 | -0,287 |
| O94903 | -0,161 | 0,207 | -0,389 | 0,166 | -0,756 | -0,723 | -0,856 | -0,706 | -0,086 | -0,115 | -0,196 | -0,931 | -0,707 | -0,898 | -1,150 | -0,262 | -0,980 | -0,470 | -0,236 | 0,142 |
| Q9NRV9 | -0,463 | 0,697 | -0,095 | -0,259 | -0,410 | 0,406 | 0,151 | -0,162 | -0,418 | -0,754 | -0,068 | -0,317 | 0,065 | -0,227 | -1,285 | -0,172 | -0,929 | -0,204 | -0,811 | 0,053 |
| O43491 | -0,546 | 0,985 | -0,513 | -0,553 | 0,249 | -0,345 | -1,162 | -0,458 | -0,395 | -0,357 | 0,223 | -0,624 | -0,699 | -0,683 | -1,288 | -0,198 | -1,456 | 0,431 | -0,308 | 0,368 |
| P62937 | -0,215 | -0,232 | 0,277 | -0,082 | -1,409 | 0,276 | -0,604 | -1,154 | 0,288 | -0,648 | -0,323 | 0,461 | 0,075 | -0,041 | -1,360 | -0,014 | -1,565 | -0,443 | -0,077 | -0,132 |
| P31937 | -0,384 | 0,505 | -0,663 | -0,535 | -0,624 | 0,035 | -0,879 | -0,474 | -0,635 | -0,052 | -0,152 | -0,379 | -0,345 | -0,542 | -0,471 | 0,070 | -0,137 | -1,114 | -0,897 | 0,080 |
| Q13510 | -0,203 | 0,130 | -0,830 | -0,581 | -0,169 | 0,182 | -0,490 | -0,764 | -0,167 | -0,655 | 0,113 | -0,108 | -0,218 | -0,540 | -0,469 | 0,431 | -0,797 | 0,096 | -0,255 | 0,175 |
| P35237 | -0,116 | 0,937 | -0,411 | -0,474 | -0,547 | -0,051 | -0,564 | -0,952 | -0,461 | -0,048 | -0,070 | -0,525 | -0,145 | -0,922 | -0,248 | 0,197 | -0,177 | -0,200 | 0,133 | 0,045 |
| Q96CX2 | 0,313 | 0,321 | -0,191 | -0,257 | -0,342 | -0,159 | -0,556 | -0,794 | 0,345 | -0,309 | 0,226 | -0,536 | -0,445 | -0,555 | -0,364 | -0,504 | -0,323 | -0,272 | 0,078 | 0,153 |
| Q66K74 | -0,034 | 0,136 | -0,276 | 0,111 | -0,782 | -0,502 | 0,598 | -0,630 | -0,436 | -0,425 | -0,342 | -0,292 | -0,090 | -0,632 | -0,396 | -0,205 | -0,550 | -0,743 | -0,365 | -0,780 |
| P25786 | 0,060 | -0,155 | 0,109 | 0,035 | -0,615 | -0,013 | -0,616 | -0,210 | -0,105 | -0,658 | -0,113 | 0,007 | -0,456 | -0,375 | -0,642 | -0,166 | -0,815 | 0,172 | -0,368 | -0,211 |
| Q9UBW8 | -0,392 | 0,863 | -0,481 | -0,277 | -0,807 | -0,138 | -0,651 | -0,629 | -0,277 | -0,382 | 0,088 | -0,242 | -0,088 | -0,528 | -0,568 | -0,012 | -0,597 | 0,049 | -0,836 | -0,133 |
| Q9BZ67 | 0,476 | -0,033 | -0,832 | -0,537 | -0,653 | -0,569 | 0,058 | 0,057 | 0,034 | 0,256 | 0,288 | -0,363 | -0,395 | -0,939 | -0,165 | -0,407 | -0,265 | -0,074 | 0,366 | 0,118 |
| P31153 | 0,237 | -0,055 | 0,132 | 0,366 | -1,155 | -0,047 | -0,632 | -0,131 | 0,049 | -0,003 | -0,117 | -0,293 | -0,105 | -0,133 | -1,115 | -0,216 | -1,176 | -0,194 | -0,255 | -0,398 |
| P36915 | 0,297 | 0,288 | -0,397 | 0,205 | -0,890 | -0,585 | -0,045 | -0,026 | 0,200 | -0,287 | -0,185 | 0,019 | -0,319 | -0,020 | -0,431 | -0,743 | -0,294 | 0,264 | 0,109 | -0,025 |
| Q13618 | -0,220 | -0,215 | -0,106 | -0,006 | -0,415 | -0,133 | 0,333 | 0,210 | 0,602 | -0,225 | 0,038 | 0,072 | -0,144 | 0,065 | -0,181 | -0,310 | -0,295 | -0,539 | -0,349 | -0,234 |
| P46781 | -0,542 | 0,124 | -0,564 | -0,123 | -0,411 | -0,905 | 0,075 | 0,033 | -0,315 | -0,519 | -0,831 | 0,155 | -0,731 | -0,732 | -0,929 | -0,103 | -1,427 | -0,262 | -0,812 | -0,325 |
| P50402 | -0,553 | 0,532 | -0,769 | -0,281 | -0,403 | -0,012 | -0,100 | -0,057 | -0,147 | -0,588 | -0,294 | -0,849 | -0,666 | 0,363 | -0,337 | -0,773 | -0,093 | -0,512 | -0,805 | -0,314 |
| Q9Y5S2 | -0,110 | 0,415 | -0,821 | 0,181 | -0,170 | 0,654 | 0,272 | -0,236 | -0,397 | 0,495 | -0,303 | 0,291 | -0,528 | -0,138 | -0,118 | 0,002 | 0,114 | -0,079 | -0,030 | -0,079 |
| P16152 | -0,339 | 0,544 | -0,177 | -0,069 | -0,841 | -0,526 | -0,606 | -0,253 | -0,047 | 0,093 | 0,209 | -0,110 | 0,150 | -0,505 | -0,509 | -0,314 | -0,686 | -0,518 | -0,045 | 0,086 |
| P09960 | -0,216 | 0,149 | -0,275 | -0,052 | -0,417 | -0,110 | -0,341 | -0,756 | -0,207 | -0,079 | -0,208 | -0,507 | 0,566 | -0,612 | -0,247 | 0,017 | -0,184 | -0,128 | 0,185 | -0,620 |
| O43488 | -0,340 | 0,477 | -0,259 | 0,228 | -0,393 | -0,508 | -0,642 | -0,621 | -0,146 | -0,658 | 0,087 | -0,296 | -0,302 | -0,448 | -0,445 | -0,023 | -0,319 | -0,488 | -0,223 | -0,331 |
| Q07812 | 0,085 | -0,144 | 0,043 | -0,084 | -0,105 | -0,374 | -0,024 | -0,200 | 0,308 | -0,087 | 0,467 | 0,509 | 0,028 | -0,226 | -0,007 | -0,069 | -0,750 | 0,436 | -0,028 | 0,215 |
| O43837 | 0,196 | 0,352 | 0,694 | 0,009 | 0,201 | 0,081 | 0,051 | 0,508 | 0,935 | 0,658 | 0,596 | 0,081 | 0,733 | 0,225 | 0,185 | 0,437 | 0,248 | 0,108 | 0,225 | 0,240 |
| Q96T58 | 0,413 | -0,112 | 0,356 | 0,272 | 0,508 | -0,270 | 0,147 | 0,248 | 0,631 | 0,475 | 0,525 | 0,162 | 0,289 | 0,563 | 0,589 | 0,203 | 0,453 | 0,081 | 0,086 | 0,016 |
| O75976 | 0,259 | -0,154 | 0,187 | 0,183 | 0,061 | 0,099 | 0,236 | 0,122 | 0,361 | 0,492 | 0,400 | 0,940 | 0,256 | 0,233 | 0,191 | 0,375 | 0,016 | 0,288 | 0,441 | 0,463 |
| O94804 | -0,410 | -0,152 | 0,207 | 0,072 | 0,855 | 0,562 | 0,375 | -0,074 | 0,390 | 0,212 | 0,322 | 0,183 | 0,565 | 0,212 | 0,779 | 0,170 | 0,278 | 0,426 | 0,067 | 0,568 |
| Q9NPA0 | 0,604 | -0,068 | 0,153 | 0,173 | 0,284 | 0,125 | 0,810 | 0,045 | 0,190 | 0,319 | 0,510 | 0,128 | 0,189 | 0,522 | 0,488 | 1,114 | 0,937 | 0,200 | -0,010 | -0,543 |
| Q96DI7 | 0,277 | 0,348 | 0,100 | 0,189 | 0,947 | 1,104 | 0,279 | -0,103 | 0,185 | -0,113 | -0,520 | 0,201 | -0,130 | 1,038 | 0,311 | -0,190 | 0,202 | 0,062 | 0,377 | 0,647 |
| O15460 | 0,229 | -0,422 | 0,125 | 0,019 | 0,045 | -0,185 | 0,191 | 0,069 | 1,498 | 0,278 | 0,353 | 0,710 | 0,528 | -0,283 | -0,177 | -0,507 | -0,299 | 0,679 | 0,126 | 0,274 |
| Q9Y6Y8 | 0,019 | 0,070 | 0,203 | -0,131 | 0,894 | 0,553 | 0,442 | 0,002 | 0,084 | 0,506 | 0,437 | 0,094 | 0,400 | 0,765 | 0,634 | 1,990 | 0,686 | -0,036 | -0,193 | -0,060 |
| P26358 | 0,610 | -0,215 | 0,351 | 0,745 | 0,597 | 1,090 | 0,231 | 1,069 | 1,112 | 0,273 | 0,103 | 0,598 | -0,251 | 1,817 | 0,094 | -0,414 | 0,200 | 0,147 | -0,034 | -0,166 |
| Q86VB7 | 0,150 | 0,153 | 0,006 | -0,123 | 2,401 | 0,463 | 0,345 | 0,076 | 0,049 | 0,550 | 0,324 | -0,080 | 0,251 | 0,131 | 0,559 | -0,053 | 0,453 | 1,521 | 0,265 | 0,650 |
| P41218 | 0,706 | -0,529 | 0,370 | -0,346 | 1,240 | -0,187 | -0,285 | -0,428 | 0,173 | 0,223 | 0,047 | -0,273 | 1,564 | -0,241 | 0,491 | 0,177 | 0,291 | 0,890 | 0,093 | 0,478 |
| P02786 | -0,181 | -0,085 | 0,349 | 0,105 | -0,524 | -0,588 | 0,937 | 2,524 | 0,798 | 0,515 | 0,818 | 0,281 | 0,329 | 1,737 | -0,518 | -0,507 | -0,293 | -0,144 | -0,120 | -0,602 |
| Q9H4B7 | 0,302 | -0,004 | 0,520 | 0,536 | 0,228 | 0,033 | 0,071 | 1,521 | 0,459 | 0,518 | 0,304 | 0,315 | 0,407 | 0,945 | 0,173 | -0,123 | 0,547 | 2,263 | 0,347 | 1,581 |
| P18428 | -0,511 | 0,263 | 0,316 | -0,695 | 0,776 | -0,502 | 0,368 | 0,391 | -0,079 | 2,281 | 0,228 | -0,459 | 2,928 | 0,518 | 1,553 | 0,080 | 0,221 | 0,845 | 1,355 | 0,676 |
| P16157 | 0,291 | -0,796 | 2,837 | -0,373 | 0,753 | -0,269 | -0,433 | 0,679 | -0,068 | 0,304 | 0,190 | -0,471 | 0,131 | 0,468 | 0,801 | 0,068 | 1,136 | 2,599 | 0,123 | 0,186 |
| P04003 | -0,501 | -0,308 | -0,517 | -0,676 | 0,648 | 0,009 | -0,038 | -0,009 | -0,026 | 3,116 | -0,024 | -0,038 | 2,399 | 1,106 | 0,421 | -0,550 | 1,684 | 0,231 | 0,909 | 1,169 |
| P02549 | 0,336 | -0,340 | 3,107 | -0,565 | 1,298 | -0,175 | -0,615 | 0,792 | 0,046 | 0,514 | 0,217 | -0,355 | -0,190 | 0,554 | 0,810 | 0,294 | 1,461 | 2,588 | 0,230 | -0,007 |
| P01011 | -0,647 | 0,310 | -0,558 | -0,890 | -0,216 | -0,645 | -0,387 | -0,414 | -0,424 | 1,790 | -0,257 | -0,065 | 3,093 | 0,187 | -0,508 | 0,294 | -0,055 | 0,586 | 1,742 | 0,168 |
| P61626 | 0,691 | -0,016 | -0,883 | -1,044 | 0,860 | -0,099 | -1,296 | -0,982 | -0,949 | 0,619 | -0,370 | -1,032 | 1,321 | -0,950 | 1,058 | 1,046 | 0,667 | 0,996 | 1,246 | 0,253 |
| P11166 | 0,429 | -0,355 | 0,889 | -0,148 | 0,332 | 0,525 | 2,044 | 0,307 | -0,087 | 0,805 | -0,112 | -0,053 | 0,482 | 0,861 | 0,429 | 0,885 | 1,178 | 1,212 | 0,491 | -0,017 |
| P02730 | 0,241 | -0,839 | 3,534 | -0,422 | 1,228 | -0,045 | -0,564 | 0,573 | -0,832 | 0,542 | -0,314 | -0,602 | 0,387 | 0,597 | 1,244 | 0,398 | 1,895 | 2,420 | -0,168 | 0,027 |
| P02788 | -0,460 | -0,497 | -0,481 | -0,877 | -2,423 | -0,786 | -2,060 | -1,833 | -1,641 | -1,056 | -1,428 | -1,001 | 3,927 | -1,406 | -0,882 | 0,624 | -0,515 | 0,544 | -0,334 | -1,640 |
| P05164 | 0,503 | -1,510 | -0,252 | -0,657 | -0,574 | -0,441 | -1,086 | -2,222 | -2,494 | -0,513 | -0,789 | -0,621 | 3,670 | -0,754 | -0,090 | 0,849 | 0,128 | 1,345 | 0,059 | -0,500 |
| P08246 | -0,275 | -0,166 | -1,056 | -1,200 | -1,298 | 0,097 | -0,966 | -0,996 | -1,141 | 0,934 | -0,722 | -0,965 | 3,619 | -0,665 | -0,237 | 0,575 | -0,035 | 1,227 | 0,398 | -1,142 |
| P02671 | -1,158 | -0,708 | -1,111 | -2,374 | 0,840 | -0,448 | 0,442 | -0,148 | -0,959 | 3,355 | -0,521 | -2,113 | 0,143 | 0,759 | 2,518 | -0,012 | 1,310 | 2,852 | 3,294 | -0,161 |
| P02679 | -2,058 | -0,905 | -1,895 | -2,999 | 0,135 | -1,086 | 0,122 | -0,315 | -0,775 | 3,678 | -0,654 | -2,808 | 0,159 | 0,313 | 2,258 | -0,149 | 1,116 | 1,894 | 3,279 | -0,723 |
| P02675 | -2,196 | -1,000 | -1,842 | -2,740 | 0,319 | -0,534 | -0,021 | -0,647 | -1,084 | 3,556 | -0,730 | -2,372 | 0,108 | 0,356 | 2,260 | -0,208 | 1,185 | 2,255 | 3,648 | -0,691 |

# Feature Selection

# Supplementary Table 2: Proteins selected as a result of BORUTA variable selection for classifying residual disease after neoadjuvant chemotherapy with machine learning models

| **Group** | **Proteins Accession ID** |
| --- | --- |
| Residual disease (R0, R1) | O75874, P78417, P11766, P12955, P07305, Q14914, P00568, P49189, P48637, Q8NCW5, P48739, O43491, P35237, Q96CX2, P25786, Q96T58, O95302, P02788 |

# Evaluation Criteria

Supplementary Table 3: Classification matrix for calculating performance criteria

|  | | **Real** | | |
| --- | --- | --- | --- | --- |
|  |  | **Positive** | **Negative** | **Total** |
| **Predicted** | **Positive** | True positive (TP) | False positive (FP) | **TP+FP** |
|  | **Negative** | False negative (FN) | True negative (TN) | **FN+TN** |
|  | **Total** | **TP+FN** | **FP+TN** | **TP+TN+FP+FN** |

Accuracy = (TP+TN)/(TP+TN+FP+FN)

Sensitivity = TP/(TP+FN)

Specificity = TN/(FP+TN)

F1-score = (2*TP)/(2*TP+FP+FN)

AUC: The AUC is a performance metric that measures the area under the Receiver Operating Characteristic (ROC) curve, which is a graphical representation of a model's ability to distinguish between two classes (usually a positive class and a negative class) across different probability thresholds.

Brier Score: The Brier score is used as a metric to measure the accuracy of the model's predicted probabilities in classification problems. The Brier score measures how close the model's predicted probabilities are to the true class. The score ranges from 0 to 1, the closer to 0 the better the model's predictions. A low Brier score indicates that the model's probability predictions are both precise and accurate.
